# Drugs for spontaneous coronary dissection: a few untrusted options

**DOI:** 10.3389/fcvm.2023.1275725

**Published:** 2023-11-10

**Authors:** Ivan Ilic, Anja Radunovic, Stefan Timcic, Natalija Odanovic, Dragana Radoicic, Natasa Dukuljev, Gordana Krljanac, Petar Otasevic, Svetlana Apostolovic

**Affiliations:** ^1^ Department of Cardiology, Institute for Cardiovascular Diseases Dedinje, Belgrade, Serbia; ^2^ School of Medicine, University of Belgrade, Belgrade, Serbia; ^3^ Cardiology Clinic, University Clinical Center of Serbia, Belgrade, Serbia; ^4^ Cardiology Clinic, University Clinical Center Nis, Nis, Serbia; ^5^ School of Medicine, University of Nis, Nis, Serbia

**Keywords:** spontaneous coronary dissection, antiplatelets, beta-blocker, ACE inhibitor, statin

## Abstract

Spontaneous coronary artery dissection (SCAD) is a rare cause of acute coronary syndrome that is often overlooked, misdiagnosed, and maltreated. Medical treatment poses a significant challenge because of the lack of randomized studies to guide treatment. The initial clinical presentation should guide medical and interventional management. Fibrinolytic agents and anticoagulants should be avoided because they could favor hematoma propagation. In patients with SCAD, antiplatelet therapy should be prescribed especially dual antiplatelet therapy (DAPT) consisting of aspirin and clopidogrel, whereas potent P2Y12 inhibitors, e.g., ticagrelor and prasugrel, should be avoided. If a stent was used, DAPT should be continued for 12 months. Aspirin only can be an option for patients without “high-risk” angiographic features—thrombus burden, critical stenosis, and decreased coronary flow. Beta-blocking (BB) agents should be used to prevent recurrence of SCAD. There is a general agreement that angiotensin-converting enzyme inhibitors, angiotensin-receptor blockers, mineralocorticoid antagonists, and loop diuretics should be used in patients with SCAD experiencing the symptoms of heart failure and a decrease in left ventricular ejection fraction below 50%. Although without firm evidence, statins can be used in SCAD due to their pleiotropic properties. The results of a randomized trial on the use of BB and statins are awaited. Aggregation of data from national registries might point out truly beneficial medications for patients with SCAD.

## Introduction

Spontaneous coronary artery dissection (SCAD) is a rare cause of acute coronary syndrome (ACS) that is often overlooked, misdiagnosed, and maltreated. The missed opportunities for timely diagnosis and adequate treatment come from a relatively small proportion of all ACS patients suffering from the condition, different pathophysiological mechanisms compared to atherosclerotic ACS, non-typical ACS patients (women aged 40–60 years of age without atherosclerotic risk factors), and procedural issues regarding interventional treatment. The confusion is further amplified by the misnomer “dissection” because the condition infrequently occurs as a consequence of a tear in the intimal layer that leads to blood accumulation in the media as was previously thought (inside–out theory). The dominant mechanism would be the rupture of *vasa vasorum* leading to hematoma formation in the media that compresses the coronary artery “true” lumen (outside–in theory) (1, 2). The predisposing factors associated with the condition are female gender, peripartum period, and fibromuscular dysplasia (FMD), and the patients usually do not have traditional atherosclerotic risk factors—heredity, smoking, hypertension, diabetes, or dyslipidemia. Although it is relatively rare in the overall population of ACS patients undergoing coronary angiography (2%–4%), its incidence among ACS patients younger than 50 years of age rises to 25%. Emotional and physical stressors may contribute to the development of SCAD (2).

Knowing these facts about SCAD, it is no wonder that adequate treatment may not be easily conceived. Due to many uncertainties regarding the etiology and clinical and angiographic presentation of the disorder, medical treatment poses a significant challenge. This is further amplified by the lack of randomized studies and large registries that could generate sufficient data to guide treatment. Although timely reperfusion in the case of ST-elevation myocardial infarction (STEMI) is the ultimate goal, it may not be easily achieved in SCAD. Stent implantation may cause hematoma propagation proximal and distal from the initial site and further compromise the lumen. On the other hand, SCAD can occur in multiple coronary artery territories, so the decision about the appropriate treatment [percutaneous coronary intervention (PCI) or surgery] may be perplexing (3).

## Antiplatelet therapy in SCAD treatment

In patients presenting with ACS, the currently recommended medical treatment consists of dual antiplatelet therapy (DAPT) with aspirin and a potent P2Y12 receptor inhibitor (4). However, this therapy could cause harm in patients with SCAD, unless this included stent implantation. Based on the pathophysiology of the disease, medical treatment should aim for the preservation of flow in the affected artery and cessation of hematoma propagation, which may be conflicting goals to achieve. Fibrinolytic therapy has been shown to extend the dissection and worsen prognosis in these patients (3).

The reason for DAPT use in patients with SCAD may be caused by an idea to achieve platelet inhibition to prevent thrombosis, which is in concert with current guidelines for ACS patients in general (4). In a Swiss cohort of 107 patients with SCAD, 90% of them received DAPT consisting of aspirin and clopidogrel in 51% of cases and aspirin and ticagrelor in 40% of cases, despite that only a minority of them underwent revascularization [seven PCI and one coronary bypass grafting (CABG)] (5). However, imaging studies using optical coherence tomography (OCT) failed to demonstrate a significant thrombus burden in patients with SCAD. In patients where hematomas were fenestrated and communicated with true lumen, the incidence of thrombus on OCT was little more than 30%, while in the so-called “non-fenestrated” cases, the thrombus was seen in only 14% of cases (1). Knowing the etiology of SCAD and the findings of imaging studies, the role of thrombus formation in this entity is probably not very important. On the other hand, ACS as a condition provokes prothrombotic mechanisms regardless of the causative mechanism of coronary artery occlusion and flow impairment. The narrowing of the vessel itself activates inflammatory and immune mechanisms that could further aggravate vessel thrombosis regardless of the initial pathway that led to coronary ischemia and ACS (6). This may be the rationale for continuing DAPT in SCAD patients. Although a relatively small registry by Feldbaum et al. (7) has demonstrated that increased use of DAPT, the expanding knowledge regarding the etiology of SCAD, and more frequent use of intravascular imaging, which led to the foundation of the non-atherosclerotic nature of the disease, could provide more evidence that the less aggressive antiplatelet regimen could be equally effective in this type of ACS. The importance of this topic and the lack of available evidence was recognized by the European Society of Cardiology (ESC), which has included it in the “gaps in evidence” of the recently published guidelines on ACS. It has been suggested that the way to overcome this issue would be to start a randomized trial to evaluate different antithrombotic strategies in patients with SCAD (4). Due to the relatively small proportion of these patients in the ACS population that are frequently unrecognized, we will have to wait for a while before reaching the recommendation for the use of antithrombotic agents in these patients.

One of the relatively large registries of SCAD patients including 23 centers in Italy and Spain has found that DAPT may be harmful to these patients. The “DIssezioni Spontanee COronariche (DISCO)” registry included 314 patients where 199 were treated conservatively, of which 67 (33.7%) were treated with single platelet therapy (SAPT) and 132 (66.3%) were prescribed DAPT. Mostly, DAPT consisted of aspirin and clopidogrel (63%), while 38% of patients were prescribed a combination of aspirin and a potent P2Y12 inhibitor ticagrelor. In the SAPT group, aspirin was given in 93% of patients and ticagrelor in 6% of patients. After 1 year, DAPT was associated with a higher rate of major adverse cardiac events (MACE) compared to SAPT (18.9% vs. 6.0%; HR 2.62, 95% CI 1.22–5.61, *P* = 0.013). The difference in MACE rate was mostly due to non-fatal myocardial infarction (15.2% vs. 3.0%; HR 3.20, 95% CI 1.33–7.69, *P* = 0.009) and unplanned PCI (12.1% vs. 1.5%; HR 3.69, 95% CI 1.36–9.91, *P* = 0.01). Most of the events occurred within 1 month after initial hospital admission, and in the multivariable analysis, the prescribed DAPT was an independent predictor of events and was associated with more than four times higher risk of MACE (HR 4.54; 95% CI 1.31–14.28; *P* = 0.016). Interestingly, bleeding events were neither very frequent in any group, nor was DAPT associated with higher bleeding rates (8). The higher incidence of MACE can be explained by early hematoma propagation and further aggravation of ischemia that required intervention in the DAPT group where a significant proportion of patients received potent P2Y12 inhibitors (2). Also, the most frequent type of SCAD in this registry was type 2, both 2A and 2B, which encompassed around 60% of the cases in both groups. This type can further aggravate more significant stenosis and ischemia due to hematoma propagation under potent DAPT. Noteworthy, the SAPT group had more type 4 SCAD (SAPT 26.9 vs. DAPT 16.0%), which will not probably progress and cause new adverse events if left untreated (8). Other large registries have not found this association between antiplatelet therapy and MACE events. In a Swiss registry, almost 50% of patients received DAPT consisting of aspirin and ticagrelor or prasugrel. There was no difference in the MACE rate regarding the type of antiplatelet therapy prescribed (5).

A large Canadian registry that included 750 patients did not demonstrate adverse events related to the use of DAPT during 3 years of follow-up, although more than 80% of patients were treated conservatively. Over 90% were treated with aspirin, and 67.4% received clopidogrel or any other adenosine diphosphate (ADP) antagonist at hospital discharge. Interestingly in this large cohort, SCAD was confirmed using intracoronary imaging [intravascular ultrasound (IVUS), OCT] in less than 10% of cases, whereas 63.8% had preserved thrombolysis in myocardial infarction (TIMI) flow grade III at diagnostic coronary angiogram (9, 10).

When it comes to antiplatelet treatment in SCAD ACS, another important question arises: What is the optimal duration of DAPT? There is a consensus that patients who underwent PCI with stent implantation should be treated according to guidelines for ACS—12 months of DAPT (2, 4). On the other hand, it remains unknown how long to prescribe DAPT in patients who were treated conservatively after being diagnosed with SCAD and the ones who underwent balloon angioplasty with different kinds of devices (semi- or non-compliant balloons, scoring, or cutting balloons).

**Figure 1:**
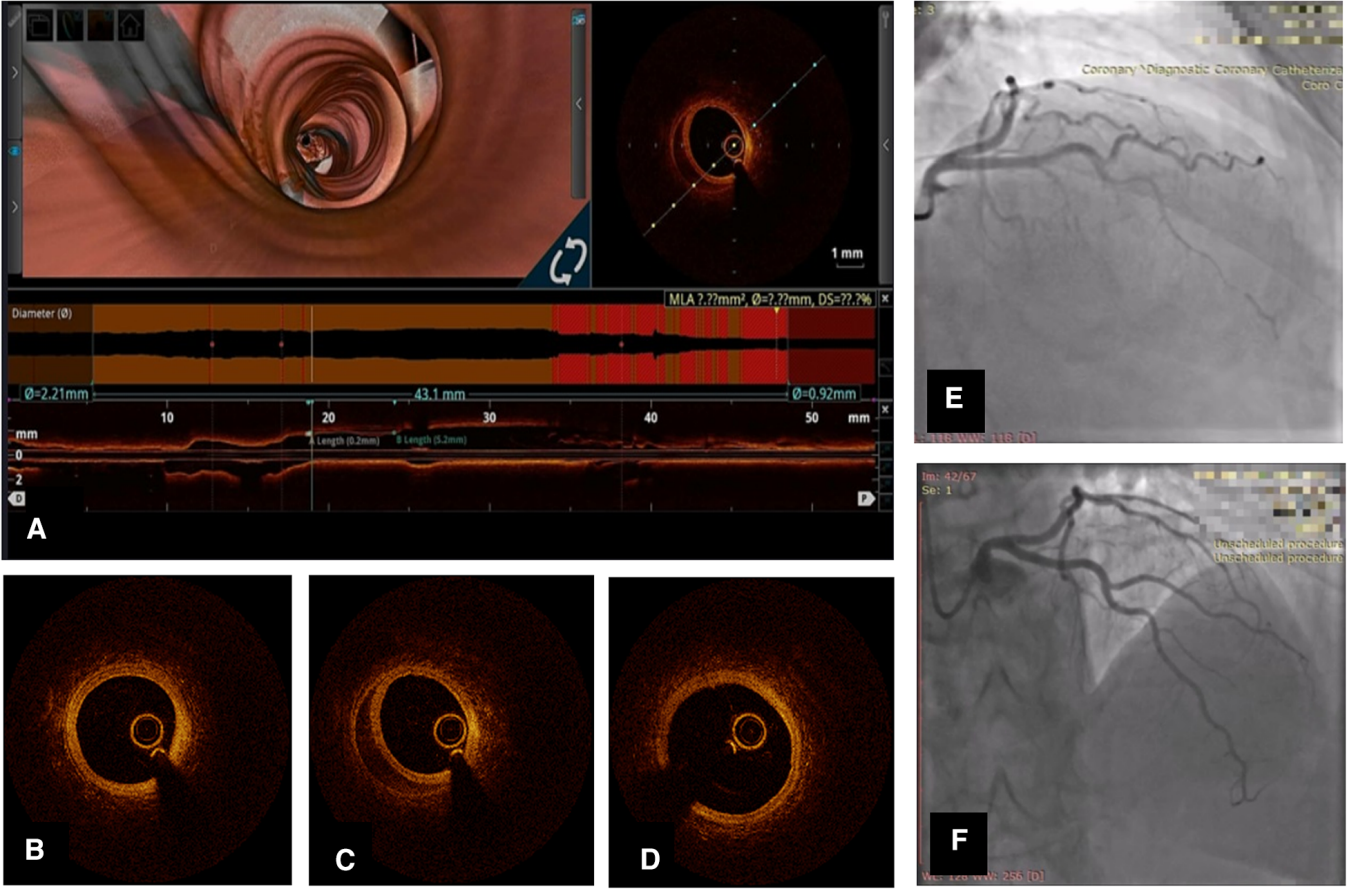
Coronary angiography and OCT of left anterior descending artery (LAD) SCAD in a 57-year-old lady. (**A**) Longitudinal OCT image 1 month after initial event with 3D flythrough showing persistent hematoma. (**B**) OCT cross-sectional image distal to SCAD lesion; (**C**) OCT cross-sectional at the level of hematoma; (**D**) OCT cross-section proximal to hematoma; (**E**) Coronary angiography of LAD [right anterior oblique (RAO)-cranial] at initial hospitalization; (**F**) Repeated coronary angiography 1 month later.

There is a clear trend toward spontaneous healing of SCAD lesions. We present the images of a 57-year-old female with treatment for hypertension, who presented with NSTEMI caused by SCAD in the left anterior descending territory. The patient underwent coronary angiography and was treated conservatively with DAPT consisting of aspirin and clopidogrel. Repeated coronary angiography and OCT showed angiographic healing and partial resorption of hematoma in previously healthy vessels (Figure 1).

The data from the Canadian registry, which included more than 150 patients who underwent repeated angiography on the average of 154 days (IQR 70–604 days), showed a resolution of stenosis in most cases with residual stenosis dropping to 25.5% (IQR 12.0%–38.8%) and only minority of the angiograms with decreased TIMI flow grade of less than 3—10/182 lesions (5.5%) in SCAD containing vessel. The authors stated that angiographic healing occurred in 157 of 182 lesions (86.3%). It is worth noting that angiographic healing occurred in 95% of lesions on coronary angiographies performed within 30 days of the event (11). The angiographic follow-up in patients in the Swiss registry also revealed a low incidence of persistent SCAD [3/68 patients (4%)] in the conservatively treated group that underwent angiography at a median of 6 months (IQR 5.5–6.5 months) (5). In both registries, the overall incidence of MACE was low, and in the Canadian registry post-discharge, MACE incidence was 8.4% after 3 years of follow-up, while in the Swiss registry after a median of 7.5 years, MACE events occurred in 15/105 patients (14.2%) (5, 10).

The duration of DAPT should be tailored according to the incidence and timing of MACE events in SCAD patients during follow-up. Large registries reported relatively low mortality during long-term follow-up in this group of ACS patients with estimated survival greater than 90%. However, the overall MACE rates in SCAD patients are relatively high due to recurrent spontaneous dissections and target vessel failure (TVF) after PCI (2, 5, 8–10, 12). What is notable is the high incidence of MACE during initial hospitalization and up to 1 month of follow-up (10). Repeated SCAD, whether an extension of the initial injury or a new affection in a different territory, is one of the major characteristics of the disease. Its reported incidence varies from 42 patients (5.6%) in the Canadian registry, 11/105 patients in the Swiss registry up to 17% in the US registry (5, 10, 12). All this has to be taken into account when planning an antiplatelet strategy in a SCAD patient.

Recently adopted new interventional strategies, such as balloon angioplasty using a “cutting” balloon or treatment with a thrombus aspiration catheter to induce a tear that would allow hematoma emptying, present another challenge in tailoring antiplatelet treatment of SCAD patients (13, 14). It seems reasonable to treat these patients similarly to the ones with atherosclerotic disease who underwent plain old balloon angioplasty (POBA) in the early days of interventional cardiology. The suggested regimen then was 1-month DAPT and continued aspirin afterward (15) (Table 1).

**Table 1:** Overview of registries of patients with SCAD regarding BB and antiplatelet agents.

Study (year of publication)	No. of subjects	Age (years)	Females (%)	Conservative treatment [*n* (%)]	BB [*n* (%)]	SAPT [*n* (%)]	DAPT [*n* (%)]	Clopidogrel in DAPT (%)	Other P2Y2 inhibitor [*n* (%)]	MACE [*n* (%)]	Bleeding events [*n* (%)]	Comments
Rogowski et al. (16) (2017)	64	53	94	56 (87)	55 (86)	5 (8)	59 (92)	44 (69)	15 (23)	5 (8)	NA	
García-Guimaraes et al. (17) (2021)	318	53	88	248 (78)	247 (79)	132 (41)	186 (59)	129 (41)	69 (22)	18 (6)	NA	In hospital f/up
Cerrato et al. (8) (2021)	314	52	89	199 (63)	157/199 (79)^a^	67/199 (34)^a^	132/199 (66)^a^	83/132 (63)^a^	49/132 (36)^a^	29/199 (14)^a^	2 (1)^a^	1 year f/up
Combaret et al. (18) (2021)	373	51	90	295 (79)	NA	NA	252/361 (69)	NA	NA	45 (12.3)	NA	
Seidl et al. (5) (2021)	105	53	93	97/105 (93)	83 (80)	10 (9)	95 (90)	48 (51)	47 (49)	15/105 (14)	NA	Median f/up 7.5 years
Daoulah et al. (19) (2021)	83	44	51	33 (40)	74 (89)	79 (97)	50 (61)	NA	NA	12 (14)	NA	Median f/up 18 month
Saw et al. (10) (2022)	750	51	88	(84)	632 (84)	244 (32)	505 (67)	NA	NA	105 (14)	NA	Median f/up 3 years

NA, not available.

^a^ Conservatively treated group.

## Fibrinolytics in SCAD treatment

Although fibrinolytic therapy represents an appropriate option in patients with STEMI that cannot be treated with an invasive strategy in a timely manner, it may pose a significant problem for the SCAD patients suffering from this form of ACS (4). Knowing the nature of the hematoma formation and the possible absence of visible thrombus on intravascular imaging, one could expect that giving a fibrinolytic agent in SCAD patients could be associated with hematoma propagation and new formation as we have previously documented (3). On the other hand, prolonged ischemia could cause irreversible damage to the myocardium subtended by the coronary artery affected with SCAD. Although there are case reports demonstrating the benefits of fibrinolytics in SCAD STEMI, ESC has, in the position statement, deemed fibrinolytics contraindicated in SCAD patients (2). However, it may sometimes pose a challenge to discern a SCAD patient from an atherosclerotic STEMI patient. It is on the clinician to weigh the potential risks and benefits of fibrinolytic treatment in an unusual female STEMI patient without known atherosclerotic risk factors presenting with typical SCAD precipitating factors.

## Anticoagulants in SCAD treatment

Most ACS patients receive anticoagulants according to current guidelines to treat ACS. After confirmation of SCAD using intravascular imaging, there is no indication to use anticoagulants, unless needed for prevention of thromboembolic events (atrial fibrillation, deep venous thrombosis). The same works for the patients who undergo PCI in SCAD, since relevant guidelines do not recommend anticoagulation after a successful PCI procedure (2, 4). This can be applied to most SCAD patients, but the ones that experience heart failure require mechanical circulatory support or mechanical ventilation and should be anticoagulated according to hospital protocols for the treatment of critically ill patients (20).

## Beta-blockers in SCAD treatment

There is conflicting evidence regarding the use of beta-blockers (BB) in patients with SCAD despite intuitively beneficial effects on blood pressure and oxygen consumption reduction that could reduce the wall shear stress and contain the propagation of dissection. The idea to use BB in SCAD patients was extrapolated from the studies in patients with aortic dissection where the use of oral and intravenous BB had profound effects on morbidity and mortality (21). However, the etiological mechanisms are sometimes quite different between these two entities, except for aortic intramural hematoma that resembles the mechanism of SCAD formation.

The study by Saw et al. (22) demonstrated that the use of BB was associated almost threefold decreased risk of recurrent SCAD in a cohort of more than 300 patients, where the incidence of recurrent SCAD was around 10% and the authors specifically excluded the patients who were perceived with extension of previously diagnosed SCAD. However, the characteristics of this study group must be taken into account when discussing the use of BB in SCAD. Most of the patients presented as non-STEMI, whereas only a quarter of them had STEMI. Regarding risk factors, one-third of them were treated for hypertension, and a quarter had dyslipidemia. The average left ventricular ejection fraction (LVEF) was 57% with 21% with LVEF lower than 50%. Most of the patients had type 2 SCAD, and more than 60% of them had normal TIMI 3 flow (22). A recently published meta-analysis confirmed the beneficial effects of BB. The analysis that included 14 studies with more than 4,000 patients found that the use of BB (HR, 0.51; 95% CI, 0.33–0.77, *P* = 0.0013) was associated with a lower risk of SCAD recurrence (23). Based on these findings, BBs were used in more than 80% of patients long term in large contemporary registries (5, 8, 10). Despite compelling evidence on BB treatment in SCAD, their use could be limited by adverse effects such as bradycardia and hypotension, which may provoke vasospasm complicating conservatively treated SCAD. In patients with SCAD affecting the right coronary artery supplying the conduction system, BBs should be used with caution (Table 1).

We are awaiting the results of the first randomized trial on the use of BB and DAPT in patients with SCAD. This ambitious study, named BA-SCAD (BB and antiplatelet agents in patients with SCAD), plans to enroll around 600 patients in a 2 × 2 factorial design and randomize them to BB (yes/no) and a short course (1 month) and long course (12 months) of DAPT (24). The study will include only patients with LVEF greater than 50% since the ones with decreased systolic function should be treated according to current guidelines for myocardial infarction that recommend BB in patients with decreased LVEF (4).

The use of BBs in SCAD should be guided by measuring the potential benefits of their use against the risks and contraindications. In addition, one should bear in mind that SCAD patients are usually BB naïve and that treatment should be carefully tailored and monitored throughout the hospital stay.

## Heart failure treatment in SCAD

There is a general agreement that angiotensin-converting enzyme (ACE) inhibitors, angiotensin-receptor blockers (ARB), mineralocorticoid antagonists, and loop diuretics should be used in patients with SCAD experiencing the symptoms of heart failure with rise in natriuretic peptides and decrease in LVEF below 50% (2, 25). The use of heart failure therapy in SCAD patients with normal LVEF cannot be justified. The use of ACE inhibitors or ARBs differs in registries of SCAD patients. In the DISCO study, there were no data on the use of heart failure medications, while in the study by Saw and associates, ACE inhibitors/ARBs were used in more than 60% of patients at discharge and more than 40% of them remained on this therapy after 3 years (8, 10). Interestingly, in the Swiss registry, the patients who experienced adverse events were less often treated with ACE inhibitors/ARBs, the difference that did not reach statistical significance [3/15 (21%) vs. 41/90 (46%); *P* = 0.09] (5). However, a SAFER-SCAD study (statin and ACE inhibitor on symptoms in patients with SCAD) (NCT 02008786) might provide answers on heart failure medication use in SCAD. The study was registered in 2013, but unfortunately, there has not been a paper published on this design yet. The purpose was to measure invasively coronary flow reserve (CFR) and index of microcirculatory resistance (IMR) in 40 SCAD patients at least 3 months after initial event and then to investigate prospectively in randomized fashion whether the addition of an ACE inhibitor or a statin to usual care in patients with ongoing chest pain and a CFR of <3.0 improves clinical status evaluated by Seattle Angina Questionnaire (SAQ) at 16 weeks compared to placebo (26). Since FMD has been frequently associated with SCAD, care should be taken when prescribing renin–angiotensin system inhibitors since there have been cases of significant renal artery stenosis associated with FMD (2, 27). These drugs should not be linearly prescribed to SCAD patients before a thorough evaluation of associated conditions that may interfere with the intended treatment.

## Statins in SCAD

Intuitively, adding a statin to the initial treatment of SCAD associated with ACS seems reasonable from the standpoint of their pleiotropic effects on inflammation and angiogenesis (28). However, simple extrapolation from atherosclerotic coronary artery lesions may not be entirely justified.

Despite the prevailing opinion that SCAD patients do not have traditional atherosclerotic risk factors, including dyslipidemia, the data from the large registries demonstrate that these patients can often suffer from this disorder. The DISCO registry revealed that 37.2% of patients had dyslipidemia prior to the SCAD event (8). On the other hand, in the largest registry from Canada, only 20.3% were diagnosed with lipid disorders (10). Surprisingly, there were more than 60% of patients with SCAD from the Swiss cohort who were diagnosed with lipid disorder with an average low-density cholesterol (LDL) of 3.3 ± 0.9 mmol/L (5). If you decide to add statin to the medical treatment of SCAD patients, another question arises—what are the target levels of LDL that we want to achieve in SCAD? Should we follow the guidelines for ACS mdash;“strike early and strike strong”—or should we initiate moderate-intensity statin and then adjust therapy according to the obtained results (29)? We might get some of the answers to these questions from the results of randomized trials of BA-SCAD and SAFER-SCAD, but still, some of the dilemmas on statin use in SCAD ACS will remain.

## How to treat SCAD medically?

Before presenting an opinion on the optimal medical treatment of patients with SCAD, it is important to disclose that there is still no randomized data regarding any medical treatment of SCAD. Our suggestion will refer only to patients with confirmed SCAD on imaging study (OCT or IVUS). Due to the low recurrence of SCAD, therapy might be limited to a shorter period of time compared to atherosclerotic ACS.

Antiplatelet agents should be prescribed in patients with SCAD, especially DAPT consisting of aspirin and clopidogrel and limited to 1 month based on the high incidence of recurrence of intimal tear in this period after the initial event. In patients presenting with large thrombus burden, after balloon angioplasty with non-compliant or cutting balloon, it seems reasonable to prescribe DAPT consisting of aspirin and clopidogrel also for 1 month. However, in patients without “high-risk features” such as concomitant atherosclerosis, large thrombus burden, critical stenosis that was left untreated, and significant flow impairment in affected coronary arteries, it would be prudent to prescribe SAPT consisting of only aspirin. If the affected vessel was treated with stent implantation, DAPT should be prescribed according to the guidelines for up to 12 months after the event. The potent P2Y12 inhibitors such as ticagrelor or prasugrel should be avoided because the potential benefits of powerful platelet inhibition would be offset by the risk of hematoma propagation.

Anticoagulation should not be routinely prescribed in SCAD patients unless indicated for thromboembolic prevention of deep vein thrombosis (DVT) or in patients having an episode of atrial fibrillation. In addition, anticoagulation should be used, according to hospital protocols, in patients who experience cardiogenic shock and are mechanically ventilated or treated with mechanical circulatory support.

Based on available data, BB agents should be used in treating SCAD patients. Caution should be employed when starting BB therapy due to the risk of bradycardia and hypotension. The therapy may be started using intravenous formulations of metoprolol or esmolol initially and then switching to oral preparations and long-acting agents with dose titration. BB should be continued for at least 6–12 months bearing in mind the time interval necessary for spontaneous healing of SCAD lesions.

If heart failure develops, it is indicated to start ACE inhibitors/ARBs, mineralocorticoid receptor antagonists, and diuretics according to current guidelines. Since FMD is frequently associated with SCAD, in patients diagnosed with FMD presenting with SCAD, care should be taken to exclude renal artery stenosis when using ACE inhibitors/ARBs. In addition, usual care is necessary to avoid hypotension, volume depletion, and worsening of renal function when starting heart failure medications in SCAD patients.

**Figure 2:**
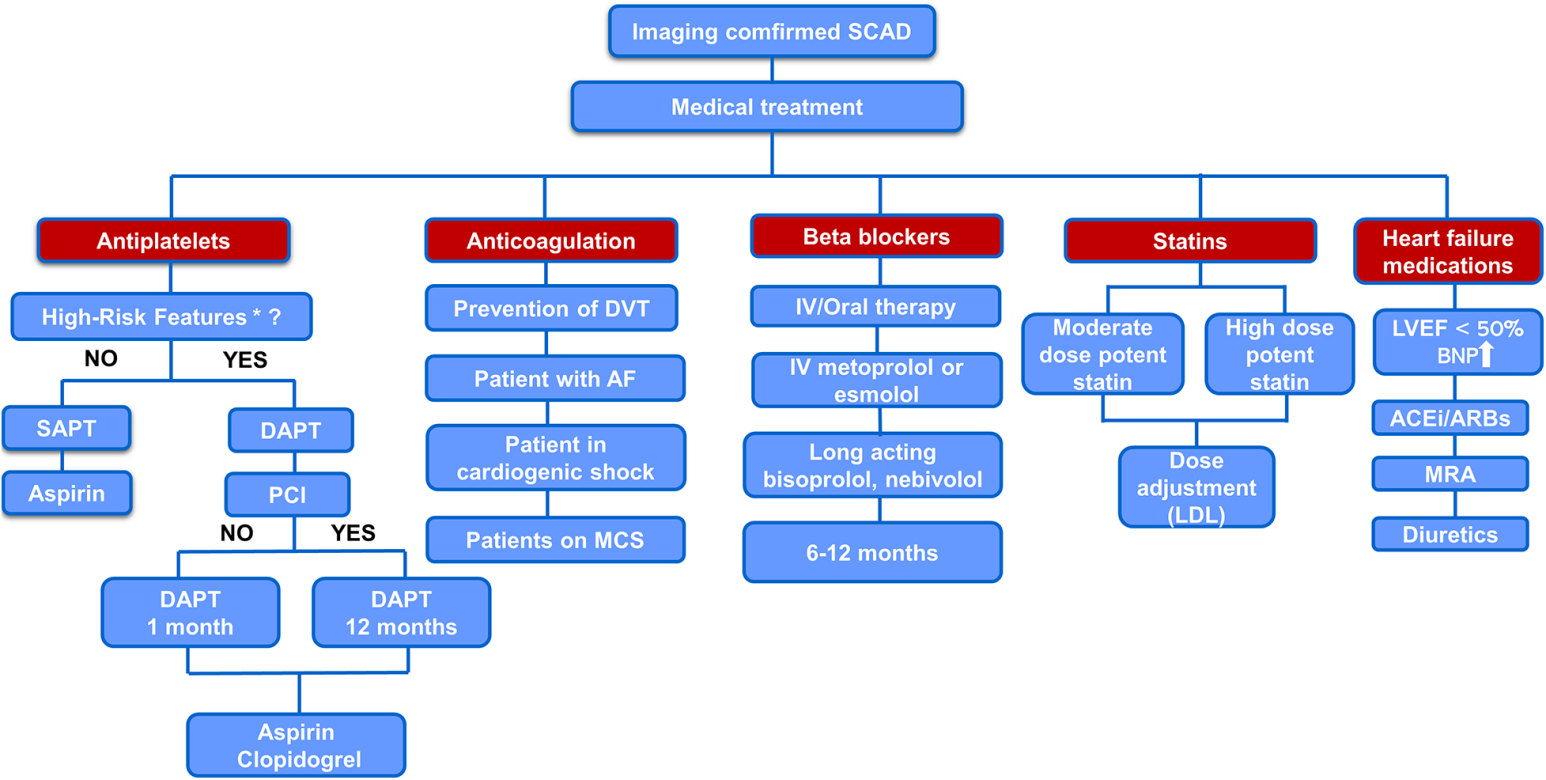
Proposed algorithm for medical treatment of imaging confirmed SCAD. *“high-risk” features—concomitant atherosclerosis, large thrombus burden, critical stenosis that was left untreated, and significant flow impairment in the affected coronary artery. AF, atrial fibrillation; BNP, brain natriuretic peptide; IV, intravenous; MCS, mechanical circulatory support; MRA, mineralocorticoid receptor antagonist.

There is no clear evidence on the use of statins in SCAD. Based on the available data regarding the effects of statins on inflammation and angiogenesis in SCAD and bearing in mind the low incidence of adverse events associated with lipid-lowering medications, we advocate selective lipid lowering with statins in SCAD patients, possibly high doses with close monitoring of the effects and adverse events (Figure 2).

Finally, it should be stated that optimal medical therapy for SCAD may not be easy to find despite the accumulation of evidence about its course and knowledge on disease pathophysiology. Probably, it will not be possible to have a randomized study that would encompass every aspect of medical treatment for this disease. Aggregation of data from national registries might point out truly beneficial medications for patients with SCAD.
